# Protein-protein interaction prediction based on multiple kernels and partial network with linear programming

**DOI:** 10.1186/s12918-016-0296-x

**Published:** 2016-08-01

**Authors:** Lei Huang, Li Liao, Cathy H. Wu

**Affiliations:** 1 Department of Computer and Information Sciences, University of Delaware, 18 Amstel Avenue, Newark, Delaware, 19716 USA; 2 Center for Bioinformatics and Computational Biology, University of Delaware, 15 Innovation Way, Newark, Delaware, 19711 USA

**Keywords:** Protein interaction network, Network inference, Interaction prediction, Random walk, Linear programming

## Abstract

**Background:**

Prediction of *de novo* protein-protein interaction is a critical step toward reconstructing PPI networks, which is a central task in systems biology. Recent computational approaches have shifted from making PPI prediction based on individual pairs and single data source to leveraging complementary information from multiple heterogeneous data sources and partial network structure. However, how to quickly learn weights for heterogeneous data sources remains a challenge. In this work, we developed a method to infer *de novo* PPIs by combining multiple data sources represented in kernel format and obtaining optimal weights based on random walk over the existing partial networks.

**Results:**

Our proposed method utilizes Barker algorithm and the training data to construct a transition matrix which constrains how a random walk would traverse the partial network. Multiple heterogeneous features for the proteins in the network are then combined into the form of weighted kernel fusion, which provides a new "adjacency matrix" for the whole network that may consist of disconnected components but is required to comply with the transition matrix on the training subnetwork. This requirement is met by adjusting the weights to minimize the element-wise difference between the transition matrix and the weighted kernels. The minimization problem is solved by linear programming. The weighted kernel fusion is then transformed to regularized Laplacian (RL) kernel to infer missing or new edges in the PPI network, which can potentially connect the previously disconnected components.

**Conclusions:**

The results on synthetic data demonstrated the soundness and robustness of the proposed algorithms under various conditions. And the results on real data show that the accuracies of PPI prediction for yeast data and human data measured as AUC are increased by up to 19 % and 11 % respectively, as compared to a control method without using optimal weights. Moreover, the weights learned by our method Weight Optimization by Linear Programming (WOLP) are very consistent with that learned by sampling, and can provide insights into the relations between PPIs and various feature kernel, thereby improving PPI prediction even for disconnected PPI networks.

## Background

Protein-protein interaction (PPI) plays an essential role in many cellular processes. In order to have a better understanding of intracellular signaling pathways, modeling of protein complex structures and elucidating various biochemical processes, many high-throughput experimental methods, such as yeast two-hybrid system and mass spectrometry method, have been used to uncover protein interactions. However, these methods are known to be prone to having high false-positive rates, besides their high cost. Therefore, great efforts have been made to develop efficient and accurate computational methods for PPI prediction.

Many pair-wise biological similarity based computational approaches have been developed to predict if any given pair of proteins interact with each other, based on various properties such as sequence homology, gene co-expression, phylogenetic profiles, three-dimensional structural information, etc. [1–7]. However, without first principles to tell deterministically if two given proteins interact or not, the pair-wise biological similarity based on various features and attributes can run out its predictive power, as the signals may be weak, noisy, or inconsistent, which can present serious issues even for methods based on integrated heterogeneous pair-wise features, e.g. genomic features, semantic similarities, etc. [8–11].

To circumvent the limitations with using pair-wise biological similarity, pair-wise topological features have been used to measure the similarity for any given node pair to make PPI prediction for the corresponding proteins [12–15], if a PPI network is constructed with nodes representing proteins and edges representing interactions. Moreover, to go beyond these node centric topological features and get the whole network structure involved, variants of random walk [16] based methods [17–19] have been developed, but the computational cost of these methods increases by *N* times for all-against-all PPI prediction. Thus many kernels on network for link prediction and semi-supervised classification have been systematically studied [20], which can measure the random-walk distance for all node pairs at once. But both the variants of random walk and random walk based kernels do not perform well in detection of interacting proteins when the direct edge connecting them in the network is removed and the remaining path connecting them is long [20]. Besides, instead of computing proximity measures between nodes from the network structure explicitly, many latent features based on rank reduction and spectral analysis have been utilized to do prediction, such as geometric de-noise methods [1, 21], multi-way spectral clustering [22], matrix factorization based methods [23, 24]. Mostly, the prediction task of these methods will be reduced to the convex optimization problem whose objective function should be carefully designed to ensure fast convergence and avoid being stuck in the local optima. Furthermore, biological features and topological features can supplement each other to improve the prediction performance, such as by assigning weights to edges in the network based on pair-wise biological similarity scores. Then, methods based on explicit or latent features, such as supervised random walk [19] or matrix factorization method, can be applied to the weighted network to make prediction, based on multi-modal biological sources. [23, 24]. However, for these methods, only the pair-wise features for the existing edges in the PPI network will be utilized, even though from a PPI prediction perspective what is particularly useful is to incorporate pair-wise features for node pairs that are not currently linked by a direct edge but will if a new edge (PPI) is predicted.

Therefore, it is of great interest if we can infer PPI network directly from multi-modal biological features kernels that involve all node pairs. It not only can help us improve prediction performance but also provide insights into relations between PPIs and various similarity features of protein pairs. Yamanishi et al. [25] developed a method based on kernel canonical correlation analysis to infer PPI networks from multiple types of genomic data. However, in that work all genomic kernels are simply added together, with no weights to regulate these heterogeneous and potentially noisy data sources for their contribution towards PPI prediction. Meanwhile, it seems that the partial network needed for supervised learning based on kernel CCA need to be sufficiently large, e.g., a leave-one-out cross validation is used, to attain good performance. In Huang et al. [26] the weights for different data sources are optimized using a sampling based method, ABC-DEP, which is computationally demanding.

In this paper, we propose a new method to infer de novo PPIs by combining multiple data sources represented in kernel format and obtaining optimal weights based on random walk over the existing partial network. The novelty of the method lies in the use of Barker algorithm to construct the transition matrix for the training subnetwork and find the optimal weights by linear programing to minimize the element-wise difference between the transition matrix and the adjacency matrix, aka, the weighted kernel from multiple heterogeneous data. Then we apply regularized Laplacian kernel (RL) to the weighted kernel to infer missing or new edges in the PPI network. A preliminary version of this work was described in [27]. Relative to that paper, the current work includes extension to handle interaction prediction problem for PPI networks consisting of disconnected components and new results on the human PPI network, which is much more sparse than the yeast PPI network. Our method can circumvent the issue of unbalanced data faced with many machine learning methods in bioinformatics by training on only a small partial network. Our method works particularly well with detecting interactions between nodes that are far apart in the network.

## Methods

### Problem definition

Formally, a PPI network can be represented as a graph *G*=(*V,E*) with *V* nodes (proteins) and *E* edges (interactions). *G* is defined by the adjacency matrix *A* with *V*×*V* dimension:

1 $$ {A(i,j)} = \left\{ \begin{array}{c} 1, if {(i,j)}\in{E} \\ 0, if {(i,j)}\notin{E} \\ \end{array} \right.  $$

where *i* and *j* are two nodes in the nodes set *V*, and (*i,j*) represents an edge between *i* and *j*, (*i,j*)∈*E*. The graph is called *connected* if there is a path of edges to connect any two nodes in the graph. Given many PPI networks are not connected and has many connected component with various size, we select a large connected component (e.g. largest connected component) as golden standard network to do supervised learning. Specifically, by adopting the same setting in [26], we divide the golden standard network into three parts: connected training network *G*_*tn*_=(*V,E*_*tn*_), validation set *G*_*vn*_=(*V*_*vn*_,*E*_*vn*_) and testing set *G*_*tt*_=(*V*_*tt*_,*E*_*tt*_), such that *E*=*E*_*tn*_∪*E*_*vn*_∪*E*_*tt*_, and any edge in G can only belong to one of these three parts.

A kernel is a symmetric positive definite matrix *K*, whose elements are defined as a real-valued function *K*(*u,v*) satisfying *K*(*u,v*)=*K*(*u,v*) for any two proteins *u* and *v* in the data set. Intuitively, the kernel built from a given dataset can be regarded as a measure of similarity between protein pairs with respect to the biological properties, from which kernel function takes its value. Treated as an adjacency matrix, a kernel can also be thought of as a complete network in which all the proteins are connected by weighted edges. Kernel fusion is a way to integrate multiple kernels from different data sources by a linear combination. For our task, this combination is made of the connected training network and various feature kernels *K*_*i*_, *i*=1,2,3...*n*, which formally is defined by Eq. (2)

2 $${} K_{fusion} = W_{0}G_{tn} + \sum\limits_{i=1}^{n} W_{i}K_{i},\ where\ K_{i}(u,v) = \frac{K_{i}(u,v)}{\sum\limits_{w}K_{i}(u,w)}  $$

Note that the training network is incomplete, i.e., with many edges taken away and reserved as testing examples. Therefore, the task is to infer or recover the interactions in the testing set *G*_*tt*_ based on the kernel fusion. Once the kernel fusion is obtained, it will be used to make PPI inference, in the spirit of random walk. However, instead of directly doing random walk, we apply regularized Laplacian (RL) kernel to the kernel fusion, which allows for PPI inference on the whole network level. The regularized Laplacian kernel [28, 29] is also called the normalized random walk with restart kernel in Mantrach et al. [30] because of the underlying relations to the random walk with restart model [17, 31]. Formally, it is defined as Eq. (3), where *L*=*D*−*A* is the Laplacian matrix made of the adjacency matrix *A* and the degree matrix *D*, and 0<*α*<*ρ*(*L*)^−1^ and *ρ*(*L*) is the spectral radius of *L*. Here, we use kernel fusion in place of the adjacency matrix, generating a regularized Laplacian matrix *RL*_*K*_, so that various feature kernels in Eq. (2) are incorporated in influencing the random walk with restart on the weighted networks [19]. With the regularized Laplacian matrix, no random walk is actually needed to measure how "close" two nodes are and then use that closeness to infer if the two corresponding proteins interact. Rather, *RL*_*K*_ is interpreted as a probability matrix *P* in which *P*_*i,j*_ indicates the probability of an interaction for protein *i* and *j*.

3 $$ RL = \sum\limits_{k=0}^{\infty} \alpha^{k}{(-L)}^{k} = {(I+\alpha*L)}^{-1}  $$

To ensure good inference, it is important to learn optimal weights for *G*_*tn*_ and various *K*_*i*_ to build kernel fusion *K*_*fusion*_. Otherwise, given the multiple heterogeneous kernels from different data sources, the kernel fusion without optimized weights is likely to generate erroneous inference on PPI.

### Weight optimization with linear programming (WOLP)

Given a PPI network, the probability of interaction between any two proteins is measured in terms of how likely a random walk in the network starting at one node will reach the other node. Here, instead of solely using the adjacency matrix *A* to build the transition matrix, we integrate kernel features as edge strength. Then the stochastic transition matrix *Q* can be built by:

4 $$ {Q(i,j)} = K_{fusion}(i,j)  $$

Assuming the network is reasonably large, for a start node *s*, the probability distribution *p* of reaching all nodes via random walk in *t* steps can be obtained by applying the transition matrix *Qt* times:

5 $$ p^{t} = Q^{t} p^{0}  $$

where the initial distribution *p*^0^ is

6 $$ {{p^{0}_{i}}} = \left\{ \begin{array}{l} 1, if \ i = s \\ 0, otherwise \\ \end{array} \right.  $$

The stationary distribution *p*, when letting *t* go to infinity, is obtained by solving the following eigenvector equation:

7 $$ p = Q~ p  $$

This stationary distribution provides constraints at optimizing the weights. For example, the positive training examples (nodes that are closer to the start node *s*) should have higher probability than the negative training examples (nodes that are far away from *s*). In Backstrom et al. [19], this is used as constraint in minimizing the L2 norm of the weights for optimal weights. In the work of Backstrom et al. [19], a gradient descent optimization method is adopted to get optimal weights, and only the pair-wise features for the existing edges in the network are utilized, which means *Q*(*i,j*) is nonzero only for edge (*i,j*) that already exists in the training network. To leverage more information from multiple heterogeneous sources, in our case the *Q*(*i,j*), as defined in Eq. (4), are nonzero unless there is no features for edge *i,j* in all kernels *K*_*a*_. Having many non-zero elements in *Q* makes it much more difficult for the traditional gradient descent optimization method to converge and to find the global optima.

In this work, we propose to solve the weights optimization differently. We can consider the random walk with restarts process shown in Eq. (5) as a Markov model, with a stationary distribution *p*. Knowing the stationary distribution, the transition matrix can be obtained by solving the reverse eign problem using the well-known Metropolis algorithm or Barker algorithm. In this work, we adopt Barker algorithm [32], which gives the transition matrix as follows.

8 $$ Q^{b}(i,j) = \frac{p_{j}}{p_{i}+p_{j}}  $$

Now we can formulate weights optimization by minimizing the element-wise difference between *Q*^*b*^ and *Q*. Namely,

9 $$ W^{*}= \mathop{argmin}_{W}|| Q - Q^{b}||^{2}  $$

As the number of elements in the transition matrix is typically much larger than the number of weights, Eq. (9) provides more equations than the number of variables, making it an overdetermined linear equation system. This overdetermined linear equation system can be solved with linear programming using standard programs in [33, 34].

**Fig. 1:**
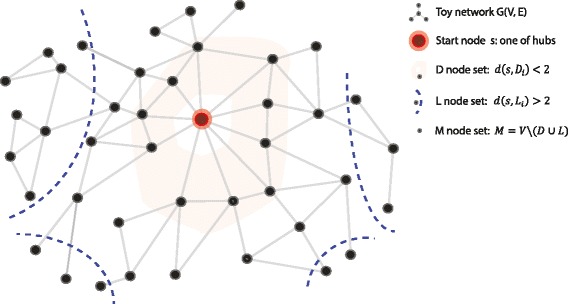
Schematic illustration of node sets D, M and L, with respect to source node s

Now, in the spirit of supervised learning, given the training network *G*_*tn*_ and a start node *s*, we calculate *p*^′^ by doing random walk that start at *s* in *G*_*tn*_ as an approximation of *p*, and $Q^{b}(i,j) = \frac {p'_{j}}{p'_{i}+p'_{j}}$. Note that *Q*^*b*^(*i,j*) from Barker algorithm is an asymmetric matrix whereas *Q* composed from kernel fusion is a symmetric matrix. So, we do not need to use all equations obtained from Eq. (9) to calculate the weights. Instead we can just use equations derived from the upper or lower triangle part of the matrices *Q*^*b*^ and *Q*. This reduction of number of equations will not pose an issue as the system is overdetermined; rather this will help mitigate the issue of being overdetermined. Specifically, as shown in Fig. 1, for all destination nodes *V* in *G*_*tn*_, namely reachable from start node *s*, we divide them into three subsets *D*, *L* and *M*, where *D* consists of near neighbors of *s* in *G*_*tn*_ with the shortest path between *s* and nodes *D*_*i*_ satisfying *d*(*s,D*_*i*_)<*ε*1; and *L* includes faraway nodes of *s* in *G*_*tn*_ with the shortest path between *s* and nodes *L*_*i*_ satisfying *d*(*s,L*_*i*_)>*ε*2; and the rest of nodes are in subset *M*. Then the system of equations of Eq. (9) is updated to Eq. (10), where *u*<*v* indicates lower triangle mapping, and *u*>*v* indicates upper triangle mapping.

10 $$ \small \begin{array}{l} W_{0}G_{tn}(u,v) + \sum\limits_{i=1}^{n} W_{i}K_{i}(u,v) = Q^{b}(u,v), \\ if\ {{u,v} \in {D \cup L}\ \land \ {K_{i}(u,v) != 0}} \land {(u<v \lor u>v)}\\ \end{array}  $$

The optimized weights *W*^∗^ can then be plugged back into Eq. (4) to form an optimal transition matrix for the whole set of nodes, and the random walk from the source node using this optimal transition matrix hence leverages the information from multi data sources and is expected to give more accurate prediction for missing and/or de novo links: nodes that are most frequented by random walk are more likely, if not yet detected, to have a direct link to the source node. The formal procedure for solving this overdetermined linear system and inferring PPIs for a particular node is shown by Algorithm 1.



### PPI prediction and network inference

As we discussed in introduction section, the use of random walk from a single start node is not efficient for all-against-all prediction, especially for the large and sparse PPI networks. Therefore, it would be of great interest if the weights learned by WOLP based on a single start node can also work network wide. Actually, it is widely observed that the many biological networks contain several hubs (i.e., nodes with with high degree) [35]. Thus we extend our algorithm to all-against-all PPI inference by hypothesizing that the weights learned based on a start node with high degree would be utilizable by other nodes. We will verify this hypothesis by doing all-against-all PPI inference for real PPI network.

We design a supervised WOLP version that can learn weights more accurately for the large and sparse PPI network. Similarly, if the whole PPI network is connected, then the golden standard network is itself; otherwise, the golden standard network that used to do supervised learning should be a large component of the disconnected PPI network. To do so, we divide the golden standard network into three parts: connected training network *G*_*tn*_=(*V,E*_*tn*_), validation set *G*_*vn*_=(*V*_*vn*_,*E*_*vn*_) and testing set *G*_*tt*_=(*V*_*tt*_,*E*_*tt*_), such that *E*=*E*_*tn*_∪*E*_*vn*_∪*E*_*tt*_, and any edge in G can only belong to one of these three parts. Then we use WOLP to learn weights based on *G*_*tn*_ and *G*_*vn*_, and finally use *G*_*tt*_ to verify the prediction capability of these weights. The main structure of our method is shown by Algorithm 2, and the supervised version of WOLP is shown by Algorithm 3. The *while* loop in Algorithm 3 is used to find optimal setting of *D*, *L* and mapping strategy(upper or lower) that can generate best weights *W*_*opt*_ with respect to inferring and *G*_*tn*_ and *G*_*vn*_.





Moreover, many existing network-level link prediction or matrix completion methods [1, 19, 21, 23, 24] can only work well on connected PPI networks, but detection of interacting pairs for disconnected PPI networks has been a challenge for these methods. However, our WOLP method can solve the problem effectively. Because various feature kernels can connect all the disconnected components of the originally disconnected PPI network; and we believe once the optimal weights have been learned based on the training network generated from a large connected component (e.g. largest connected component), they can also be used to build the kernel fusion when the prediction task scale up to the originally disconnected PPI network. To do so, we update the Algorithm 2 to Algorithm 4 that shows the detailed process of interaction prediction for disconnected PPI networks. Given an originally disconnected network *G*, firstly, we learn the optimal weights by Algorithm 3 based on a large connected component *G*_*cc*_ of *G*. After that, we randomly divide the edge set *E* of the disconnected *G* into training edge set *G*_*tn*_ and testing edge set *G*_*tt*_, and use the optimal weights we learned before directly to linearly combine *G*_*tn*_ and other corresponding feature kernels to build the kernel fusion, and finally evaluate the performance through predicting *G*_*tt*_. Here we call *G*_*tn*_ training edge set, because *G*_*tn*_ no longer needs to be connected to learn any weights.



## Results and discussion

We examine the soundness and robustness of the proposed algorithms with use of both synthetic and real data. Our goal here is to demonstrate that the weights obtained by our method can help build a better kernel fusion leading to more accurate PPI prediction.

### Experiments on single start node and synthetic data

A synthetic scale-free network *G*_*syn*_ with 5,093 nodes is generated by Copying model [36]: *G*_*syn*_ starts with three nodes connected in a triad. Remaining nodes have been added one by one with exactly two edges for each. For instance, when a node u is added, two edges (*u,v*_*i*_),*i*=1,2 between *u* and existing nodes *v*_*i*_ will be added accordingly. Node *v*_*i*_ is randomly selected with probability 0.8, and otherwise *v*_*i*_ is selected with probability proportional to its current degree. The parameters we chose is to guarantee *G*_*syn*_ has similar size and density to DIP yeast PPI network [37] that we will use to do PPI inference later. Then we build eight synthetic feature kernels for *G*_*syn*_. The feature kernels can be classified into three categories: 3 noisy kernels, 4 positive kernels and a mixture kernel, which are defined by Eq. (11)

11 $$ {{}\begin{aligned} \left\{ \begin{array}{l} K_{noise} = R_{5093} + (R_{5093}+\eta).*{rand}_{diff}(J_{5093}, G_{syn}, \rho_{i}) \\ \\ K_{postive} = R_{5093} + (R_{5093}+\eta).*{rand}_{sub}(G_{syn}, \rho_{i})\\ \\ K_{mixture} = R_{5093} + (R_{5093}+\eta).*{rand}_{sub}(G_{syn}, \rho_{i})\\ \qquad + (R_{5093}+\eta).*{rand}_{diff}(J_{5093}, G_{syn}, \rho_{i})\\ \end{array} \right. \end{aligned}}  $$

Where *R*_5093_ indicates a 5093 by 5093 random matrix with elements between [ 0,1], which can also be seen asbackground noise matrix; *J*_5093_ indicates a 5093 by 5093 all-one matrix, *rand*_*diff*_(*J*_5093_,*G*_*syn*_,*ρ*_*i*_) is used to randomly generate a difference matrix (if (i, j) = 1 in *G*_*syn*_ and (i, j) should be 0 in the difference matrix) between *J*_5093_ and *G*_*syn*_ with density *ρ*_*i*_; *rand*_*sub*_(*G*_*syn*_,*ρ*_*i*_) is used to generate a subnetwork from *G*_*syn*_ with density *ρ*_*i*_; *ρ*_*i*_ are different for each kernel; *η* is a positive parameter between [ 0,1] and *R*_5093_ will be rebuilt every time for each kernel.

The general process of experimenting with synthetic data is: we generate synthetic network *G*_*syn*_, synthetic feature kernels *K* firstly, and then divide nodes *V* of *G*_*syn*_ into *D*, *L* and *M*, where *D* and *L* can be seen as training nodes, *M* can be seen as testing nodes. By using *G*_*syn*_, start node *s* and *K*, we can get the stationary distribution *p* based on the optimized kernel fusion $ K_{OPT} = W_{0}G_{syn}(u,v) + \sum \limits _{i=1}^{n} W_{i}K_{i}(u,v) $. Finally, we try to prove that *K*_*OPT*_ is better than the control kernel fusion $ K_{EW} = G_{syn} + \sum \limits _{i=1}^{n}K_{i} $ built by equal weights, if the *p*(*M*) is more similar to *p*^′^(*M*) based on *G*_*syn*_, as compared to *p*^″^(*M*) based on the control kernel fusion *K*_*EW*_, where *p*(*M*) indicates the rank of stationary probabilities respect to the testing node *M*. We evaluate the rank similarity between pairs (*p*(*M*),*p*^′^(*M*)) and (*p*^″^(*M*),*p*^′^(*M*)) by discounted cumulative gain (DCG) [38].

We carry out 10 experiments, each time we select one of the oldest 3 nodes as start node, and rebuild synthetic kernel *K*. In Table 1, the results show that DCG@20 between *p*(*M*) and *p*^′^(*M*) is consistently higher than that between *p*^″^(*M*) and *p*^′^(*M*) in all 10 experiments, indicating that the optimal weights *W* obtained by WOLP can help us build optimized kernel fusion that with better prediction capability, as compared to the control kernel fusion.

**Table 1:** DCG@20 of rank comparison

Repetition	DCG@20(*p*(*M*),*p* ^′^(*M*))	DCG@20(*p* ^″^(*M*),*p* ^′^(*M*))
1	0.7101	0.6304
2	0.9305	0.4423
3	0.4035	0.2657
4	0.8524	0.5690
5	0.7256	0.4417
6	0.3683	0.3009
7	0.7707	0.2753
8	1.0034	0.3663
9	0.7119	0.4603
10	0.6605	0.6123

### Experiments on network inference with real data

We use the yeast PPI network downloaded from DIP database (Release 20150101) [37] and the high-confidence human PPI network downloaded from PrePPI database [39] to test our algorithm.

#### Data and kernels of yeast PPI networks

For the yeast PPI network, some interactions without Uniprotkb ID have been filtered out in order to do name mapping and make use of genomic similarity kernels [40]. As a result, the originally disconnected PPI network contains 5093 proteins and 22,423 interactions. The largest connected component consists of 5030 proteins and 22,394 interactions, and is used to serve as the golden standard network.

Six feature kernels are included in PPI inference for the yeast data. *G*_*tn*_: *G*_*tn*_ is the connected training network that provides connectivity information. It can also be thought of as a base network to do the inference. *K*_*Jaccard*_ [41]: This kernel measure the similarity of protein pairs *i,j* in term of $\frac {neigbors(i) \cap neighbors(j)}{neighbors(i) \cup neighbors(j)}$. *K*_*SN*_: It measures the total number of neighbors of protein *i* and *j*, *K*_*SN*_=*neighbors*(*i*)+*neighbors*(*j*). *K*_*B*_ [40]: It is a sequence-based kernel matrix that is generated using the BLAST [42]. *K*_*E*_ [40]: This is a gene co-expression kernel matrix constructed entirely from microarray gene expression measurements. *K*_*Pfam*_ [40]: Similarity measure derived from Pfam HMMs [43]. All these kernels are normalized to the scale of [0,1] in order to avoid bias.

#### Data and kernels of human PPI networks

The originally disconnected human PPI network has 3993 proteins and 6669 interactions, which is much sparser than the yeast PPI network. The largest connected component that serve as the golden standard network contains 3285 proteins and 6310 interactions.

Eight feature kernels are included in PPI inference for the human data. *G*_*tn*_: *G*_*tn*_ is the connected training network that provides connectivity information. It can also be thought of as a base network to do the inference. *K*_*Jaccard*_ [41]: This kernel measure the similarity of protein pairs *i,j* in term of $\frac {neigbors(i) \cap neighbors(j)}{neighbors(i) \cup neighbors(j)}$. *K*_*SN*_: It measures the total number of neighbors of protein *i* and *j*, *K*_*SN*_=*neighbors*(*i*)+*neighbors*(*j*). *K*_*B*_: It is a sequence-based kernel matrix that is generated using the BLAST [42]. *K*_*D*_: It is a domain-based similarity kernel matrix measured by the method of neighborhood correlation [44]. *K*_*BP*_: It is a biological process based semantic similarity kernel measured by Resnik with BMA [45]. *K*_*CC*_: It is a cellular component based semantic similarity kernel measured by Resnik with BMA [45]. *K*_*MF*_: It is a molecular function based semantic similarity kernel measured by Resnik with BMA [45].

#### PPI inference based on the largest connected component

For cross validation, like in [26], the golden standard PPI network (largest connected component) is randomly divided into three parts that are connected training network *G*_*tn*_, validation edge set *G*_*vn*_ and testing edge set *G*_*tt*_, where *G*_*vn*_ is used to find optimal weights for feature kernels and *G*_*tt*_ is used to evaluate the inference capability of our method. The Table 2 shows detailed division for yeast and human PPI networks.

**Table 2:** Division of golden standard PPI networks

Species	*G*_*tn*_	*G*_*vn*_	*G*_*tt*_
Yeast	*V,E*={5,030,5,394}	*V,E*={−,1,000}	*V,E*={−,16,000}
Human	*V,E*={3,285,3,310}	*V,E*={−,300}	*V,E*={−,2,700}

With the weights learned by WOLP and using *i*_*th*_ hub as the start node, we build the kernel fusion *WOLP-K-i* by Eq. (2). PPI network inference is made by RL kernel Eq. (3), and named as *RL*_*WOLP-K-i*_, *i*=1,2,3. The performance of inference is evaluated by how well the testing set *G*_*tt*_ is recovered. Specifically, all node pairs are ranked in decreasing order by their edge weights in the RL matrix, and edges in the testing set *G*_*tt*_ are labeled as positive and node pairs with no edges in *G* are labeled as negative. An ROC curve is plotted for true positive v.s. false positives, by running down the ranked list of node pairs. To make comparison, besides the PPI inferences *RL*_*WOLP-K-i*_, *i*=1,2,3 learned by our WOLP, we also include other two PPI network inferences: $ {RL}_{G_{tn}} $ and *RL*_*EW-K*_, where $ {RL}_{G_{tn}} $ indicates RL based PPI inference is solely from the training network *G*_*tn*_, and *RL*_*EW-K*_ represents RL based PPI inference is from kernel fusion built by equal weights, e.g. *w*_*i*_=1, *i*=0,1...*n*. Additionally, *G*_*set*_∼*n* indicates there is *n* number of edges in the set *G*_*set*_, e.g. *G*_*tn*_∼5394 means the connected training network *G*_*tn*_ contains 5394 edges.

**Fig. 2:**
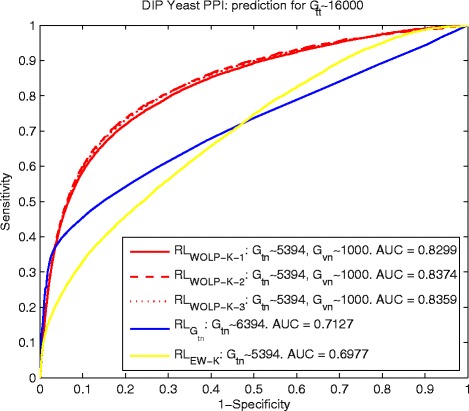
Yeast: ROC curves of predicting *G*_*tt*_∼16000 by $ {RL}_{G_{tn}\sim 5394} $, *R* *L*_*WOLP-K-i*_ and *R* *L*_*EW-K*_

**Fig. 3:**
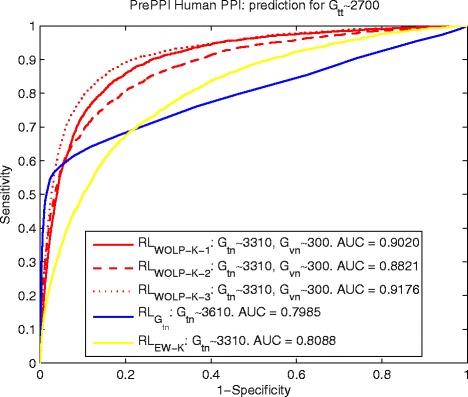
Human: ROC curves of predicting *G*_*tt*_∼2700 by $ {RL}_{G_{tn}\sim 3610} $, *R* *L*_*WOLP-K-i*_ and *R* *L*_*EW-K*_

The comparisons in terms of ROC curve and AUC for yeast and human data are shown by Fig. 2 and 3, the PPI reference *RL*_*WOLP-K-i*_, *i*=1,2,3 based on our WOLP method significantly outperforms the two basic control methods, with about 17 *%* increase over $ {RL}_{G_{tn}} $ and about 19.6 *%* over *RL*_*EW-K*_ in term of AUC for the yeast data, and about 12.7 *%* increase over $ {RL}_{G_{tn}} $ and about 11.3 *%* over *RL*_*EW-K*_ in term of AUC for the human data. It is noted that the AUC of PPI inference *RL*_*EW-K*_ based on the equally weighted built kernel fusion is no better or even worse than that of $ {RL}_{G_{tn}} $ based on a really small training network, especially for the yeast data. It means there should be a lot of noises if we just naively combine different feature kernels to do PPI prediction.

Besides inferring PPI network by using weights learned based on the top three hubs in *G*_*tn*_, we also test the predicting capability of PPI inferences by using top ten hubs as start nodes to learn the weights. We make 10 repetitions for the whole process: generating *G*_*tn*_, choosing *i*_*th*_, *i*=1,2,...10 hub as start node to learn the weights, then using these weights to build kernel fusion and finally to do the PPI inference. For the results based on top ten hubs in each repetition, the average AUC of inferring *G*_*tt*_ for yeast data and human data are shown in Tables 3 and 4 respectively. And the comparison shows the predicting capability of our method is consistently better than that of $ {RL}_{G_{tn}} $ and *RL*_*EW-K*_ for both yeast and human data.

**Table 3:** Comparison of AUCs for yeast PPI prediction

Rep	Avg AUC(*R* *L*_*WOLP-K-*1∼10_)	AUC(${RL}_{G_{tn}}$)	AUC(*R* *L*_*EW-K*_)
1	0.8367 ± 0.0134	0.7127	0.6976
2	0.7937 ± 0.0584	0.7768	0.7014
3	0.7802 ± 0.0545	0.7732	0.7009
4	0.7811 ± 0.0507	0.7406	0.7029
5	0.8349 ± 0.0301	0.7477	0.6991
6	0.8160 ± 0.0492	0.7180	0.7091
7	0.7670 ± 0.0636	0.7513	0.6992
8	0.8018 ± 0.0539	0.7739	0.7042
9	0.7989 ± 0.0552	0.7302	0.7017
10	0.8172 ± 0.0388	0.7387	0.6953

**Table 4:** Comparison of AUCs for human PPI prediction

Rep	Avg AUC(*R* *L*_*WOLP-K-*1∼10_)	AUC(${RL}_{G_{tn}}$)	AUC(*R* *L*_*EW-K*_)
1	0.8871 ± 0.0122	0.8228	0.7823
2	0.8986 ± 0.0144	0.8106	0.8127
3	0.8988 ± 0.0088	0.8216	0.8088
4	0.8955 ± 0.0114	0.8161	0.8142
5	0.8994 ± 0.0089	0.8190	0.8088
6	0.8875 ± 0.0182	0.7927	0.8067
7	0.8904 ± 0.0237	0.8302	0.8096
8	0.8978 ± 0.0121	0.8205	0.8153
9	0.9011 ± 0.0101	0.7995	0.8130
10	0.8818 ± 0.0281	0.8078	0.8104

#### Effects of the training data

Usually, given a golden standard data, we need to retrain the prediction model for different division of training set and testing set. However, if optimal weights have been found for building kernel fusion, our PPI network inference method enable us to train the model once, and do prediction or inference for different testing sets. To demonstrate that, we keep the two PPI inferences *RL*_*WOLP-K-1*_ and *RL*_*EW-K*_ obtained before (in last section) unchanged, and evaluate the prediction ability for different testing sets. We also examine how performance is affected by sizes of various sets. Specifically, while the size of training network *G*_*tn*_ for $ {RL}_{G_{tn}} $ increases, sizes of *G*_*tn*_ for *RL*_*WOLP-K-1*_ and *RL*_*EW-K*_ are kept unchanged. Therefore, we design several experiments by dividing the golden standard network into $ G_{tn}^{i} $ and $ G_{tt}^{i} $, *i*=1,...,*n*, and building PPI inference $ {RL}_{G_{tn}^{i}} $ to predict $ G_{tt}^{i} $ for every time. To make comparison, we also use *RL*_*WOLP-K-1*_ and *RL*_*EW-K*_ to predict $ G_{tt}^{i} $. As shown by the Table 5, for yeast data, *RL*_*WOLP-K-1*_ trained on only 5,394 golden standard edges still performs better than the control methods, even for the $ {RL}_{G_{tn}} $ that employ significantly more golden standard edges. Similarly, for the result of human data as shown by the Table 6, *RL*_*WOLP-K-1*_ trained on only 3,310 golden standard edges still performs better than the control method $ {RL}_{G_{tn}} $ that employ over 1,000 more golden standard edges.

**Table 5:** Effects of training data size on prediction performance (AUC) for yeast

	*G*_*tt*_∼15000	*G*_*tt*_∼14000	*G*_*tt*_∼13000
$\phantom {\dot {i}\!}{RL}_{ {WOLP-K-1}:G_{tn}\sim 5394} $	0.8658	-	-
$ {RL}_{G_{tn}\sim 7394} $	0.7931	-	-
$\phantom {\dot {i}\!} {RL}_{ {EW-K}:G_{tn}\sim 5394} $	0.7519	-	-
$\phantom {\dot {i}\!} {RL}_{ {WOLP-K-1}:G_{tn}\sim 5394} $	-	0.8659	-
$ {RL}_{G_{tn}\sim 8394} $	-	0.8538	-
$\phantom {\dot {i}\!} {RL}_{ {EW-K}:G_{tn}\sim 5394} $	-	0.7537	-
$\phantom {\dot {i}\!} {RL}_{ {WOLP-K-1}:G_{tn}\sim 5394} $	-	-	0.8659
$ {RL}_{G_{tn}\sim 9394} $	-	-	0.8619
$\phantom {\dot {i}\!} {RL}_{ {EW-K}:G_{tn}\sim 5394} $	-	-	0.7520

**Table 6:** Effects of training data size on prediction performance (AUC) for human

	*G*_*tt*_∼2600	*G*_*tt*_∼2100	*G*_*tt*_∼1600
$\phantom {\dot {i}\!} {RL}_{ {WOLP-K-1}:G_{tn}\sim 3310} $	0.9277	-	-
$ {RL}_{G_{tn}\sim 3710} $	0.8359	-	-
$\phantom {\dot {i}\!} {RL}_{ {EW-K}:G_{tn}\sim 3310} $	0.8590	-	-
$\phantom {\dot {i}\!} {RL}_{ {WOLP-K-1}:G_{tn}\sim 3310} $	-	0.9305	-
$ {RL}_{G_{tn}\sim 4210} $	-	0.8779	-
$\phantom {\dot {i}\!} {RL}_{ {EW-K}:G_{tn}\sim 3310} $	-	0.8620	-
$\phantom {\dot {i}\!} {RL}_{ {WOLP-K-1}:G_{tn}\sim 3310} $	-	-	0.9338
$ {RL}_{G_{tn}\sim 4710} $	-	-	0.9227
$\phantom {\dot {i}\!} {RL}_{ {EW-K}:G_{tn}\sim 3310} $	-	-	0.8639

#### Detection of interacting pairs far apart in the network

It is known that the basic idea of using random walk or random walk based kernels [17–20] for PPI prediction is that good interacting candidates usually are not faraway from the start node, e.g. only 2, 3 edges away in the network. Consequently, the testing nodes have been chosen to be within a certain distance range, which largely contributes to the good performance reported by many network-level link prediction methods. In reality, however, a method that is capable and good at detecting interacting pairs far apart in the network can be even more useful, such as in uncovering cross talk between pathways that are not nearby in the PPI network.

**Fig. 4:**
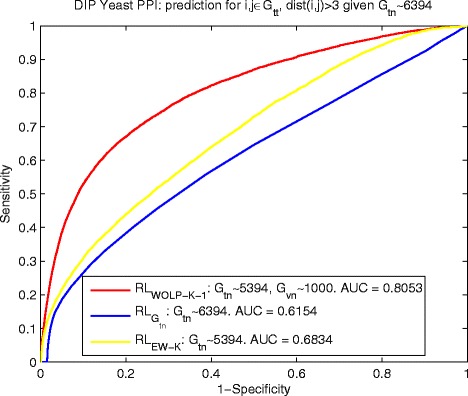
Yeast: ROC curves of predicting $ G_{tt}^{(dist(i,j)>3)} $ by $ {RL}_{G_{tn}\sim 6394} $, *R* *L*_*WOLP-K-1*_ and *R* *L*_*EW-K*_

**Fig. 5:**
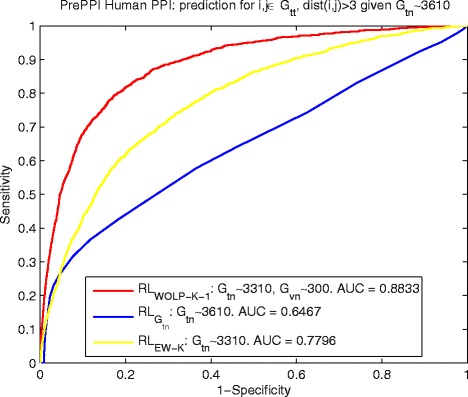
Human: ROC curves of predicting $ G_{tt}^{(dist(i,j)>3)} $ by $ {RL}_{G_{tn}\sim 3610} $, *R* *L*_*WOLP-K-1*_ and *R* *L*_*EW-K*_

To investigate how our proposed method performs at detecting faraway interactions, we still use $ {RL}_{G_{tn}\sim 6394} $, *RL*_*WOLP-K-1*_ and *RL*_*EW-K*_ for yeast data, and $ {RL}_{G_{tn}\sim 3610} $, *RL*_*WOLP-K-1*_ and *RL*_*EW-K*_ for human data to infer PPIs, but we select node pairs (*i,j*) that satisfy *dist*(*i,j*)>3 *givenG*_*tn*_ from *G*_*tt*_ as new testing set and name it $ G_{tt}^{(dist(i,j)>3)} $. Figures 4 and 5 show the results of yeast and human data respectively, which demonstrate that *RL*_*WOLP-K-1*_ has not only a significant margin over the control methods in detecting long-distance PPIs but also maintains a high ROC scores of 0.8053 (for yeast data) and 0.8833 (for human data) comparable to that of all PPIs. In contrast, in both Figs. 4 and 5, $ {RL}_{G_{tn}} $ performs poorly and worse than *RL*_*EW-K*_, which means the traditional RL kernel based on adjacent training network alone cannot detect faraway interactions well.

#### Detection of interacting pairs for disconnected PPI networks

**Fig. 6:**
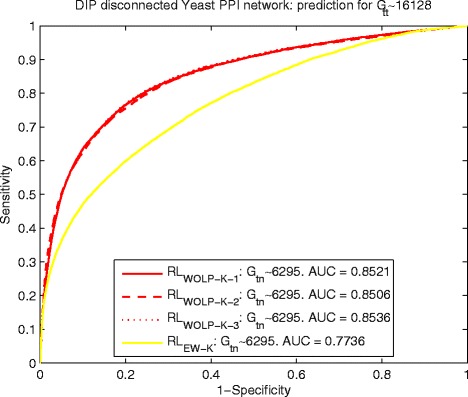
Yeast: ROC curves of predicting *G*_*tt*_∼16128 by *R* *L*_*WOLP-K-1*_ and *R* *L*_*EW-K*_ for disconnected PPI network

**Fig. 7:**
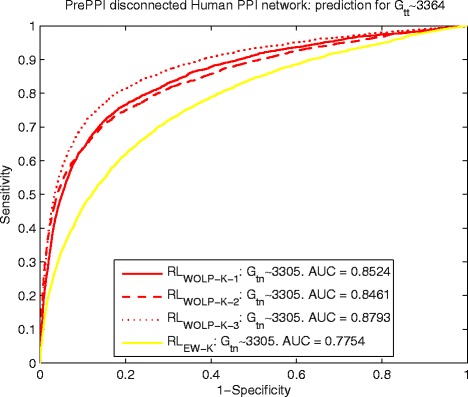
Human: ROC curves of predicting *G*_*tt*_∼3364 by *R* *L*_*WOLP-K-1*_ and *R* *L*_*EW-K*_ for disconnected PPI network

For the originally disconnected yeast PPI network, we randomly divide the edge set *E* into training edge set *G*_*tn*_ with 6295 edges and testing edge set *G*_*tt*_ with 16,128 edges. Similarity, based on a random division, the number of edges of training edge set *G*_*tn*_ and testing edge set *G*_*tt*_ are 3305 and 3364 for the originally disconnected human PPI network. The detailed information of the originally disconnected yeast and human PPI networks can be found in the subsection of data description. The Figs. 6 and 7 show the predicting results of yeast and human data respectively, which indicate *RL*_*WOLP-K-i*_, *i*=1,2,3 perform steady well on inferring interactions for both yeast and human data and are obviously better than *RL*_*EW-K*_. $ {RL}_{G_{tn}} $ is not included in this comparison, because it is not feasible for prediction tasks of disconnected PPI networks.

#### Analysis of weights

**Fig. 8:**
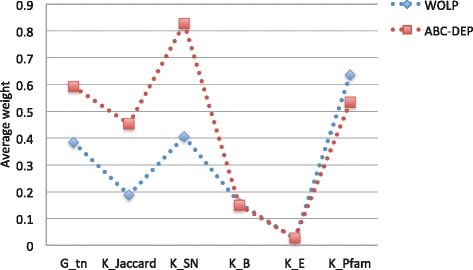
Yeast: comparison of average weights learned by WOLP and ABC-DEP sampling method

**Fig. 9:**
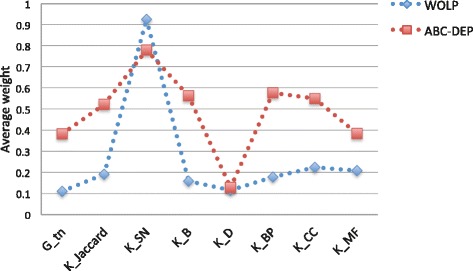
Human: comparison of average weights learned by WOLP and ABC-DEP sampling method

As our method incorporates multiple heterogeneous data, it can be insightful to inspect the final optimal weights. Therefore, we compare the average of weights learned by WOLP to the average of weights learned from revised ABC-DEP sampling method [26, 46], which is more computationally demanding. For the yeast data, the Fig. 8 shows that these two methods produce consistent results: these weights indicate that *K*_*SN*_ and *K*_*Pfam*_ are the predominant contributors to PPI prediction. This observation is consistent with the intuition that proteins interact via interfaces made of conserved domains [47], and PPI interactions can be classified based on their domain families and domains from the same family tend to interact [48–50]. For the human data, due to the extreme sparsity of the human PPI network, limited golden standard interactions can be included in the validation set to help optimize weights, which makes the weight optimization problem more challenging, especially for the sampling method. Although the result of human data that shown in Fig. 9 is not good as that of the yeast data, these two methods also produce quite consistent distribution, and *K*_*SN*_ is the most predominant contributor. Although the true strength of our method lies in integrating multiple heterogeneous data for PPI network inference, the optimal weights can serve as a guidance to select most relevant features when time and resources are limited.

## Conclusion

In this work we developed a novel and fast optimization method using linear programming to integrate multiple heterogeneous data for PPI inference problem. The proposed method, verified with synthetic data and tested with DIP yeast PPI network and PrePPI high-confidence human PPI network, enables quick and accurate inference of PPI networks from topological and genomic feature kernels in an optimized integrative way. Compared to the baseline (*G*_*tn*_ and *EW-K*), our WOLP method achieved performance improvement in PPI prediction with over 19 % higher AUC on yeast data and 11 % higher AUC on human data, and this margin is maintained even when the control methods use a significantly larger training set. We also demonstrated that by integrating topological and genomic features into regularized Laplacian kernel, the method avoids the short-range problem encountered by random-walk based methods – namely the inference becomes less reliable for nodes that are far from the start node of the random walk, and shows obvious improvements on predicting faraway interactions; The weights learned by our WOLP are highly consistent with the weights learned by sampling based method, which can provide insights into the relations between PPIs and various similarity features of protein pairs, thereby helping us make good use of these features. Moreover, we further demonstrated those relations are also maintained when the golden standard network (largest connected component) scale up to the original PPI network that consists of disconnected components. That is to say, the weights learned based on the connected training subnetwork of the largest connected component can also help to detect interactions for the originally disconnected PPI networks effectively and accurately. As more features with respect to proteins are collected from various -omics studies, they can be used to characterize protein pairs in terms of feature kernels from different perspectives. Thus we believe that our method can provide us a quick and accurate way to fuse various feature kernels from heterogeneous data, thereby improving PPI prediction.
